# Mutational landscape of epidermoid carcinoma of the penis in a Brazilian cohort

**DOI:** 10.37349/etat.2025.1002323

**Published:** 2025-06-06

**Authors:** Renato Mendes Rossi De Lucca, Danielle Barbosa Brotto, Claudia Tarcila Gomes Sares, Kelly Gomes Duarte, Wilson Araujo Silva Junior, Philippe E. Spiess, Shahrokh F. Shariat, Natália Dalsenter Avilez, Caio de Oliveira, Leonardo O. Reis, Rodolfo Borges dos Reis

**Affiliations:** IRCCS Istituto Romagnolo per lo Studio dei Tumori (IRST) “Dino Amadori”, Italy; ^1^Medicine School of Ribeirão Preto, University of São Paulo, Ribeirão Preto 14040-900, Brazil; ^2^Morsani College of Medicine, Moffitt Cancer Center, University of South Florida, Tampa, FL 33612, USA; ^3^INCT UroGen, National Institute of Science, Technology and Innovation in Genitourinary Cancer (INCT), Campinas 13087-571, Brazil; ^4^Medical University Vienna, University Hospital Vienna AKH, 1090 Vienna, Austria; ^5^Hourani Center for Applied Scientific Research, Al-Ahliyya Amman University, Amman 19328, Jordan; ^6^Department of Urology, University of Texas Southwestern Medical Center, Dallas, TX 75390, USA; ^7^Department of Urology, Weill Cornell Medical College, New York, NY 10065, USA; ^8^UroScience, School of Medical Sciences, University of Campinas (Unicamp), Campinas 13083-970, Brazil; ^9^ImmunOncology, Pontifical Catholic University of Campinas, Campinas 13060-904, Brazil

**Keywords:** Penile cancer, mutations, epidemiology

## Abstract

**Aim:**

Penile cancer (PeCa) is a rare malignancy strongly associated with poor genital hygiene and is more prevalent in regions with low socioeconomic status. PeCa accounts for approximately 2% to 4% of all male cancers in Brazil, with higher incidence in the North and Northeast regions. Despite its aggressive nature, the molecular mechanisms underlying PeCa remain poorly understood. We performed whole-exome sequencing in a Brazilian cohort of patients with PeCa to identify potentially pathogenic genetic alterations associated with tumor development and progression.

**Methods:**

Tumor tissue samples were obtained from patients diagnosed with PeCa. DNA was extracted and subjected to whole-exome sequencing. Human papillomavirus (HPV) genotyping was performed for subtypes 16 and 18. Control samples were collected from individuals without PeCa or other genital diseases.

**Results:**

The cohort demonstrated considerable genetic heterogeneity. Multiple gene mutations were identified in tumor samples, many of which are involved in carcinogenesis-related biological pathways. Distinct molecular profiles were observed, suggesting diverse tumorigenic mechanisms. *MUC16* (present in 11/12 patients, 91.7%) and *PABPC1* (8/12 patients, 66.7%) were the most frequently mutated genes. HPV-16 was detected in a subset of cases; however, no consistent association with more aggressive disease was identified.

**Conclusions:**

This study provides new insights into the genomic landscape of PeCa in a Brazilian population. The findings highlight the presence of heterogeneous and potentially pathogenic mutations, reinforcing the need for further molecular characterization and exploration of novel therapeutic targets in PeCa.

## Introduction

Among the various types of cancer affecting the male population, penile cancer (PeCa) stands out due to its relatively high mortality rate. PeCa is a rare malignancy that arises on the skin or within the tissues of the penis. The most common histological subtype is squamous cell carcinoma, which accounts for over 90% of cases and originates in the epithelial cells covering the penile surface [1].

Incidence rates of PeCa vary globally and are strongly associated with socio-economic factors. The disease is more prevalent in Latin America, Africa, and Asia [2]. In Brazil, data from the National Cancer Institute (INCA) reported 408 and 444 deaths from PeCa in 2016 and 2017, respectively [3]. According to the International Agency for Research on Cancer (IARC), there were an estimated 36,230 new cases and 15,938 related deaths worldwide in 2020 [4].

Several risk factors have been implicated in the pathogenesis of PeCa. These include chronic infection with high-risk human papillomavirus (HPV) types—especially HPV-16 and HPV-18, along with poor genital hygiene, phimosis, smoking, and absence of circumcision. Additionally, chronic inflammatory and pre-malignant conditions of the penis, such as balanoposthitis, lichen sclerosus et atrophicus, and xerotic balanitis obliterans, have been associated with increased oncogenic potential [5].

The management of PeCa is largely guided by the stage of the disease, with particular emphasis on lymph node status and the existence of metastases. For patients with localized, low-grade T1 tumors, treatment options may include conservative approaches such as local excision, partial penectomy, laser ablation, or radiotherapy. In contrast, individuals with more advanced lesions (T2 or higher) often require more aggressive interventions, including partial or total penectomy, potentially combined with radiotherapy and/or chemotherapy. In cases of non-palpable inguinal lymph nodes and low-risk primary tumors, active surveillance may be appropriate. However, in patients with intermediate- to high-risk disease (e.g., T1b or higher), non-palpable lymph nodes may necessitate evaluation through bilateral dynamic sentinel lymph node biopsy (DSNB) [6] or even bilateral inguinal lymph node dissection (ILND), followed by close follow-up [7, 8]. Palpable inguinal or pelvic lymphadenopathy typically requires confirmation via percutaneous biopsy and may be managed with pelvic lymph node dissection (PLND), radiotherapy, systemic chemotherapy, or neoadjuvant chemotherapy regimens [9, 10].

With recent advances in next-generation sequencing technologies, cancer genomics has entered a new era of discovery. The comprehensive characterization of somatic mutations in tumors has enabled the identification of novel biomarkers with potential diagnostic, prognostic, and therapeutic value. Although the oncogenic role of HPV in PeCa is still under investigation, current evidence suggests that the *E6* and *E7* viral oncogenes contribute to malignant transformation by inactivating key tumor suppressor genes, including *TP53* and *RB1*, thereby promoting uncontrolled cellular proliferation [11]. Nonetheless, HPV is not universally present in all PeCa cases, indicating that other molecular mechanisms also play a role in carcinogenesis [12].

The present study aimed to perform whole-exome sequencing of PeCa tissue from a Brazilian cohort. The objective was to identify relevant somatic mutations associated with tumor initiation or progression and to contribute genomic insights that may guide future clinical decision-making.

## Materials and methods

### Ethics statement and tissue sample collection

This study was approved by the Research Ethics Committee of the Clinical Hospital of Ribeirão Preto (University of São Paulo) under approval number 1.909.052/2016. All procedures involving human participants were performed by the ethical standards of the institutional and national research committees, as well as with the 1964 Declaration of Helsinki and its later amendments or comparable ethical standards. Written informed consent was obtained from all participants before inclusion in the study.

Tissue samples were obtained from 12 patients (P1–P12) diagnosed with PeCa who underwent either total or partial penectomy, and from six control individuals (C1–C6) undergoing postectomy, at the Clinical Hospital of the Ribeirão Preto Medical School, University of São Paulo, Brazil.

Inclusion criteria for the patient group were: male sex, age over 18 years, histologically confirmed diagnosis of PeCa, no family history of cancer, eligibility for surgical tumor resection, and no prior chemotherapy or radiotherapy. Control subjects were defined as men over 18 years of age without a diagnosis of PeCa or any known genetic or venereal disease that could compromise normal penile tissue, and who underwent postectomy for aesthetic or functional purposes.

Immediately following surgical excision, the tissue specimens were snap-frozen in liquid nitrogen and subsequently stored in the biorepository of the Urology Division at −80°C. According to the manufacturer’s instructions, genomic DNA was extracted from frozen tumor-confirmed tissue fragments using the Qiagen AllPrep DNA/RNA/miRNA Universal Kit (Qiagen, Venlo, Netherlands).

### HPV-16 and HPV-18 genotyping

The presence of HPV subtypes 16 and 18 was investigated through conventional polymerase chain reaction (PCR) amplification of genomic DNA, using specific primers listed in Table 1. PCR reactions were carried out in a final volume of 25 µL under the following thermal cycling conditions: initial denaturation at 95°C for 2 minutes; 33 cycles of denaturation at 95°C for 1 minute, annealing at 55°C for 1 minute, and extension at 72°C for 1 minute; followed by a final extension at 72°C for 7 minutes.

**Table 1:** Primers sequence for HPV genotyping

**Genotypes**	**Sequence**	**Amplicon (bp)**
HPV-16 F	5’-AGAACTGCAATGTTTCAGGACCCACAG-3’	120
HPV-16 R	5’-TCTGCAACAAGACATACATCGACCGG-3’	
HPV-18 F	5’-TGAGGATCCAACACGGC-3’	120
HPV-18 R	5’-CCTCTGTAAGTTCCAATACTGTC-3’	

F: forward primer; R: reverse primer. HPV: human papillomavirus

### Whole-exome sequencing and data processing

Whole-exome sequencing was performed on DNA samples extracted from tumor tissues. Library preparation was conducted using the Nextera Exome Enrichment Kit (Illumina Inc., San Diego, CA, USA), following the manufacturer’s protocol. Sequencing was performed with the NextSeq 500 platform (model SY-415-1001, Illumina, Inc., San Diego, California, USA) using the High Output NextSeq 500 V2 kit (Cat. No. FC-404-2003, Illumina, Inc., San Diego, California, USA) and paired-end sequencing chemistry. Raw BCL files were converted to FASTQ format using bcl2fastq (Illumina Inc., v2.20). Quality control of sequencing reads was performed using the software FastQC (v0.11.5; © 2010–2015 Babraham Bioinformatics, Babraham Institute) and summarized with MultiQC (v1.8; © 2016–2019 Phil Ewels, National Genomics Infrastructure, SciLifeLab).

Reads were aligned to the human reference genome (GRCh37/hg19) using BWA-MEM (v0.7.15; © 2009–2013 Heng Li, Broad Institute). The resulting BAM files were processed using the Genome Analysis Toolkit (GATK) (v3.8; © 2010–2019 Broad Institute) for post-alignment processing and variant calling. Functional annotation of variants was performed with snpEff (v4.3; © 2012–2017 Pablo Cingolani, Max Planck Institute for Molecular Biomedicine).

### Variant identification and annotation

Variant calling was performed using the GATK, employing the HaplotypeCaller and GenotypeGVCFs modules, followed by score recalibration. Variant annotation was conducted using snpEff (v4.2) and custom in-house Perl scripts.

To identify cancer-relevant mutations, the detected variants were cross-referenced with the Catalogue of Somatic Mutations in Cancer (COSMIC; https://cancer.sanger.ac.uk/cosmic). Variants were further filtered for predicted pathogenicity using the Combined Annotation Dependent Depletion (CADD) score (CADD > 10) and the following predictive tools: UMD-Predictor, SIFT, and PolyPhen-2.

### Statistics

Through a comparative approach involving 12 tumor samples and six non-neoplastic foreskin specimens, we provide valuable insights into the mutational landscape of PeCa. The relatively small sample size limits the generalizability of our findings and precludes robust statistical inference.

## Results

This study analyzed DNA samples extracted from the penile tissue of 18 individuals, comprising 12 patients with histologically confirmed squamous cell carcinoma of the penis and six control individuals without any clinical or histopathological evidence of PeCa, genetic disorders, or venereal disease, who underwent postectomy for aesthetic or functional reasons.

Our analysis identified exclusive somatic mutations and potential molecular biomarkers, thereby contributing to the growing body of genomic data in this rare malignancy and laying the groundwork for future studies investigating genotype-phenotype correlations and therapeutic targets.

The age at diagnosis among the cancer patients ranged from 33 to 81 years, with both the mean and median age being 57.5 years. Ethnic self-identification among these patients was as follows: 58.3% White (*n* = 7), 25.0% Black (*n* = 3), and 16.7% Mulatto (*n* = 2). A history of tobacco use was reported by 75% of patients, and 50% reported regular alcohol consumption (Table 2).

**Table 2:** Characteristics of the studied population

**Characteristics**	**Data**
Age (median)	33–81 (57.5)
Ethnic (self-declared)	
White	7 (58.33%)
Black	3 (25.00%)
Mulato	2 (16.67%)
Smoker	
Yes	9 (75.0%)
No	3 (25.0%)
Alcoholic	
Yes	6 (50.0%)
No	6 (50.0%)
Presence of HPV-16	
Positive	2 (16.67%)
Negative	10 (83.33%)
Presence of HPV-18	
Positive	0 (0.0%)
Negative	12 (100.0%)
Metastasis	
Positive	4 (33.33%)
Negative	8 (66.67%)
Surgery	
Partial penectomy	9 (75.00%)
Partial penectomy followed by total	2 (16.67%)
Total penectomy	1 (8.33%)
Death	2

HPV-16: human papillomavirus-16

Clinically, microscopic lesions suggestive of HPV infection were observed in two patients. However, molecular testing confirmed HPV-16 DNA in only one of these cases, and in one additional patient who did not exhibit visible lesions. No samples tested positive for HPV-18.

Tumor staging revealed four cases classified as pT1, three as pT2, three as pT3, and two with staging information unavailable. Among the 12 cancer patients, 4 (33.3%) presented with metastases, initially affecting regional lymph nodes. Two of these patients subsequently developed distant metastases to bone and lung and succumbed to the disease. All patients underwent surgical treatment via penectomy; nine underwent partial penectomy with tumor excision and adequate safety margins, while three underwent total penectomy.

Whole-exome sequencing and subsequent variant analysis using the COSMIC database identified potentially pathogenic variants in 55 genes. Among these, the most frequently mutated genes were *MUC16* (present in 11/12 patients, 91.7%) and *PABPC1* (8/12 patients, 66.7%). Although mutations in *PABPC1* were also detected in all control individuals, the mutation types differed: frameshift mutations were found in controls, while non-synonymous mutations were predominant in cancer patients.

**Figure 1:**
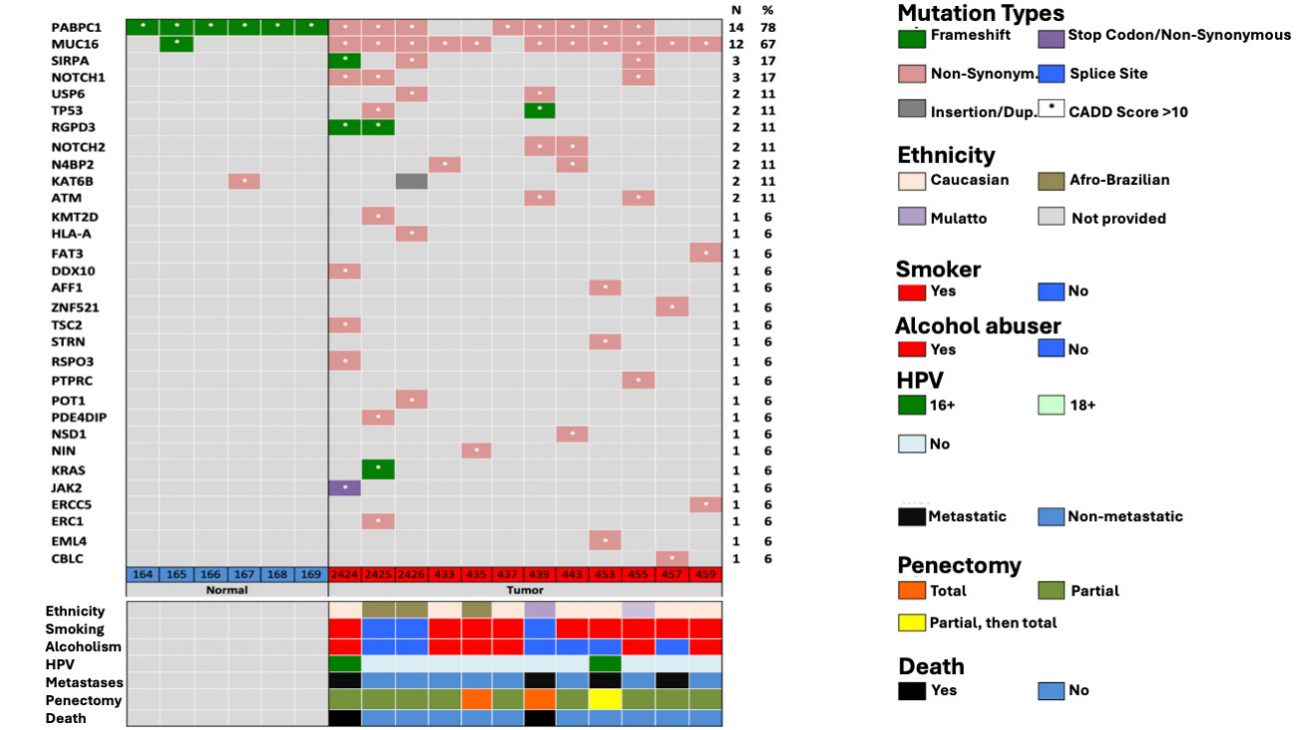
**Mutations information.** CADD: Combined Annotation Dependent Depletion; HPV: human papillomavirus

The mutation profile among patients with PeCa predominantly included non-synonymous single-nucleotide variants, followed by frameshift mutations and insertions. Other genes with notable variants include *SIRPA*, *NOTCH1*, *USP6*, *TP53*, *NOTCH2*, *KMT2D*, *ATM*, *HLA-A*, *JAK2*, *KRAS*, and several others. While some of these genes have been previously implicated in cancer, many have not yet been described in the context of penile carcinoma. A summary of the identified mutations is provided in Figure 1.

## Discussion

The therapeutic strategy for PeCa is primarily guided by disease stage, especially in cases with regional lymph node involvement or distant metastases. According to the National Comprehensive Cancer Network (NCCN) Guidelines (v1.2024), the preferred systemic treatment for patients with metastatic or recurrent disease is a regimen combining paclitaxel, ifosfamide, and cisplatin (TIP). An alternative first-line option includes cisplatin in combination with 5-fluorouracil (5-FU). In parallel, recent studies have increasingly focused on the molecular characteristics of advanced PeCa, exploring the potential of targeted therapies and immune checkpoint inhibitors based on underlying genomic alterations [10].

Through comprehensive exome sequencing, we identified several unique mutations potentially associated with tumor progression and adverse clinical outcomes.

Among the 12 patients, five exhibited distinguishing clinical characteristics: advanced local disease requiring total penectomy (P5, P7, P9), HPV positivity (P1, P9), and evidence of metastases (P1, P7, P9, P11), with two patients (P1, P7) ultimately succumbing to the disease. The remaining patients, who did not experience disease progression, served as a reference group for outlining a linear model of PeCa progression.

Patient P1, a 52-year-old Caucasian tobacco and alcohol user with HPV-16 infection, presented with advanced disease, experienced recurrence one-year post-surgery, and developed metastases to the bone and lung, ultimately leading to death. This patient harbored pathogenic mutations in *PABPC1*, *MUC16*, *SIRPA*, *NOTCH1*, *RGPD3*, *DDX10*, *TSC2*, *RSPO3*, and *JAK2*.

The tumor suppressor gene *DDX10* has been associated with inhibition of tumor growth, and its reduced expression has been implicated in accelerated cancer progression [13]. *TSC2*, a critical regulator of the mTORC1 pathway, acts as a tumor suppressor. Loss-of-function mutations in *TSC2* lead to *mTORC1* hyperactivation, stimulating anabolic processes and inhibiting autophagy [14, 15]. Notably, HPV-16 E6 proteins can bind and degrade TSC2, further activating *mTORC1* and enhancing tumor cell proliferation, metastasis, and resistance to apoptosis by inhibiting pro-apoptotic proteins such as Bad and Bax [16, 17].

The *NOTCH1* gene, frequently implicated in squamous carcinomas, was also mutated in P1. Ali et al. [18] identified *NOTCH1* mutations in 25% of PeCa samples and proposed it as a potential therapeutic target. Patient P7, diagnosed with metastatic PeCa at just 33 years of age, was a non-smoker and non-drinker who died two months after a total penectomy. This patient exhibited mutations in *TP53*, *NOTCH2*, *ATM*, and *USP6*. Although the role of *USP6* in PeCa is not well-defined, Zhou et al. [19] previously reported its frequent mutation in PeCa cell lines.

The *NOTCH2* mutation c.302G > T, a frameshift variant with a high CADD score, is predicted to be pathogenic. The *NOTCH* family genes encode transmembrane proteins involved in cellular survival and differentiation [20]. Although few studies have examined *NOTCH2* in PeCa, frequent alterations have been noted, suggesting potential relevance [19]. The *ATM* gene mutation (c.8800A > G), a non-synonymous variant, is also predicted to be pathogenic. *ATM* plays a pivotal role in genomic stability and DNA damage repair. Previous studies have linked *ATM* mutations with poor outcomes in PeCa [21, 22]. The co-occurrence of *ATM* and *TP53* mutations may disrupt DNA repair and apoptotic mechanisms, contributing to the aggressive course observed in this patient.

Patient P9, a 65-year-old Caucasian with a history of alcohol consumption, presented with HPV-16-positive, locally advanced PeCa. Mutations in *AFF1*, *STRN*, and *EML4* were identified. The *EML4*/*ALK* fusion gene has been extensively studied in lung adenocarcinoma and squamous cell carcinoma and is associated with metastasis [23]. Similarly, *STRN* mutations can generate STRN/ALK fusion proteins, previously linked to metastatic progression in colorectal, breast, and renal cancers [24–26]. These findings suggest a potential role of these genes in the lymph node metastases observed in this patient.

Patient P11, a white male tobacco user without HPV infection, presented with unilateral lymph node metastasis. Mutations were found in *ZNF521* and *CBLC*. *ZNF521* encodes a transcriptional co-repressor involved in chromatin remodeling and has been implicated in the pathogenesis of breast, lung, and colorectal cancers through its role in genomic instability [26–31]. The *CBLC* gene, affected by a non-synonymous mutation (c.1174G > A), is known to influence tumor suppression, cell cycle regulation, proliferation, and DNA repair [32, 33]. Although *CBLC* has not been widely studied in cancer, its moderate-impact mutations merit further exploration.

Interestingly, patients who underwent partial penectomy with tumor-free margins experienced no recurrence or metastasis. This subgroup, including the oldest patients and those with the highest mutation counts, suggests that neither age nor the number of mutations independently predicts disease aggressiveness. Instead, specific “driver” mutations and gene interactions may play a more decisive role. These insights support the development of targeted gene panels for identifying mutations associated with carcinogenesis and aggressive clinical behavior in PeCa.

Including healthy controls with no history of tobacco or alcohol use, penile lesions, or venereal disease allowed for the identification of shared mutations (*PABPC1*, *MUC16*, and *KAT6B*) between groups. However, controls typically harbored frameshift or isolated mutations, while cancer patients displayed non-synonymous mutations with broader genomic involvement, suggesting distinct mutation profiles. Of note, *RSPO3*—classified as pathogenic, showed no known gene interactions and was absent from the dbSNP database, suggesting it may represent a novel finding worthy of future investigation.

### Strengths and limitations

This study constitutes the largest Brazilian cohort dedicated to whole-exome sequencing of PeCa across multiple clinical stages. However, certain limitations should be considered.

Despite the originality and relevance of our exome-based investigation, as a descriptive study, we could not directly correlate the identified mutations with clinical outcomes or assess their relative prognostic significance.

PeCa is a rare yet highly aggressive malignancy that remains largely neglected, particularly because it predominantly affects populations in underdeveloped and developing countries. As a result, limited genetic studies have been conducted to elucidate the molecular mechanisms underlying its carcinogenesis.

Our study revealed substantial genetic heterogeneity within PeCa tumor tissue. Nevertheless, many of the mutated genes identified are known to play critical roles in the initiation and progression of carcinogenesis. These findings support the hypothesis that PeCa can arise and evolve through multiple disrupted pathways related to cellular homeostasis.

Additionally, the presence of HPV infection did not appear to correlate with specific mutation hotspots or increased tumor aggressiveness in our cohort. Whole-exome analysis of PeCa offers valuable insight into the genetic landscape of the disease and provides a foundational step toward identifying novel molecular markers. These findings support the development of targeted therapies and precision medicine strategies to improve clinical outcomes and survival in affected patients. The HPV-16 DNA prevalence aligns with contemporary genotyping epidemiology and poor correlations of non-molecular tests (peniscopy, cytology, and histopathology) [12].

### Future directions

Advances in exome-based sequencing have enabled the identification of genomic alterations that may guide therapy in PeCa. Both targeted treatments and immunotherapies are under active investigation for advanced or metastatic disease. One of the first reports of immune checkpoint inhibition in PeCa described a patient with chemotherapy- and radiotherapy-resistant disease who achieved a partial response with nivolumab, highlighting the potential role of immunotherapy [34].

The HERCULES trial (LACOG 0218), a phase II study conducted at 11 centers in Brazil, evaluated pembrolizumab combined with platinum-based chemotherapy in 37 patients with advanced PeCa. Among 33 patients assessed for response, 39.4% achieved objective responses, with higher benefits observed in those with high tumor mutational burden (TMB), suggesting a role for molecular markers in therapy selection [35].

Epidermal growth factor receptor (EGFR) inhibitors, such as cetuximab, and antibody-drug conjugates (ADCs) like enfortumab vedotin (nectin-4 targeted) and sacituzumab govitecan (Trop-2 targeted), are also being explored. These therapies aim to selectively deliver cytotoxic agents to tumor cells, offering a promising therapeutic avenue with potentially reduced systemic toxicity [36, 37].

## Abbreviations

CADD: Combined Annotation Dependent Depletion
COSMIC: Catalogue of Somatic Mutations in Cancer
GATK: Genome Analysis Toolkit
HPV: human papillomavirus
PCR: polymerase chain reaction
PeCa: penile cancer

## Declarations

### Availability of data and materials

The collected nonpersonal patient data and material transfer of this study are available after communication and agreement of the lead contact.

### Copyright

© The Author(s) 2025.
